# Chlorogenic Acid Ameliorates CVB3-Induced Viral Myocarditis by Suppressing Viral Replication and ZBP1-Mediated PANoptosis

**DOI:** 10.3390/microorganisms14061375

**Published:** 2026-06-21

**Authors:** Junbo Huang, Qing Song, Yanjun Di, Hao Wu, Zhiyun Cheng, Haoyi Zhan, Kaiyuan Huang, Yachen Wang, Lijuan Xie, Jieqing Liu, Lei Tong

**Affiliations:** School of Medicine, Huaqiao University, Quanzhou 362021, China; 17369909003@163.com (J.H.); 19607367883@163.com (Q.S.); 2126203012@stu.hqu.edu.cn (Y.D.); 2134133009@stu.hqu.edu.cn (H.W.); chengzhiyun@hqu.edu.cn (Z.C.); 2334111025@stu.hqu.edu.cn (H.Z.); 2534132005@stu.hqu.edu.cn (K.H.); 2434131021@stu.hqu.edu.cn (Y.W.); xielijuan@hqu.edu.cn (L.X.); liujieqing@hqu.edu.cn (J.L.)

**Keywords:** coxsackievirus B3, antiviral, Z-DNA-binding protein 1, PANoptosis, chlorogenic acid

## Abstract

Viral myocarditis (VMC), predominantly driven by Coxsackievirus B3 (CVB3) infection and the resultant excessive immune response, lacks effective treatments and specific antiviral drugs in clinical practice. Chlorogenic acid (CGA) has been proven to have significant antiviral and anti-inflammatory properties. This study evaluated the potential and mechanism of action of CGA against CVB3-induced viral myocarditis. Our research results showed that CGA significantly alleviated myocardial tissue damage in vivo. This protective effect was accompanied by effective inhibition of myocardial inflammatory responses and viral replication. Further in vitro experiments confirmed that CGA significantly inhibited the replication of CVB3 in a dose-dependent manner, and its inhibitory effect mainly targeted the replication stage of the viral life cycle. Mechanistically, CGA treatment correlates with reduced ZBP1 expression and accelerated ZBP1 degradation involving the ubiquitin–proteasome pathway, accompanied by suppressed activation of PANoptosis markers. These findings suggest that CGA alleviates CVB3-induced myocarditis through concerted antiviral and anti-inflammatory effects, with ZBP1-mediated PANoptosis as a potential contributing mechanism.

## 1. Introduction

Viral myocarditis (VMC) is a prevalent cardiovascular disorder that is characterized by direct viral invasion of the myocardial tissue, which leads to local or diffuse inflammatory damage of the myocardial parenchyma or interstitium [1,2]. The main pathological alterations of it are interstitial inflammation and necrosis or apoptosis of cardiomyocytes, which eventually can develop into an irreversible cardiac impairment [2]. The Coxsackievirus B3 (CVB3) is an enterovirus belonging to the Picornaviridae family that proves most pathogenic in VMC [3]. According to epidemiologic research, the incidence of VMC has been on the rise in recent years, and biopsy of endomyocardial tissue in about 60% of myocarditis cases has confirmed the presence of viral nucleic acids [4]. The pathogenesis is sequentially divided into acute-phase direct cytopathic effects of CVB3 replication and then CVB3-induced immune injury, which leads to the manifestation of autoimmune-mediated myocardial injury [5,6]. Unfortunately, the lack of particular antiviral agents makes clinical treatment of the VMC caused by CVB3 a serious treatment issue. As a result, the modern treatment is mainly based upon supportive care to combat the symptoms and reduce disease escalation to the heart failure stage.

PANoptosis is a proinflammatory programmed cell death pathway that integrates key molecular components of apoptosis, necroptosis, and pyroptosis into a multi-protein complex called the PANoptosome [7,8]. The PANoptosome, a multi-protein complex, coordinates inflammatory cell death by activating effector caspases (caspase-3/6/7), which trigger endonuclease-mediated DNA fragmentation and chromatin hypercondensation—hallmarks of apoptosis [9]. Upon caspase-8 blockade, RIP1 and RIP3 form the necrosome complex. [10,11]. This complex phosphorylates MLKL, causing its oligomerization and translocation to the plasma membrane, thereby inducing necroptotic death. [12,13]. Gasdermin family proteins (including GSDMD) are cleaved specifically by the inflammatory caspases (caspase-1/4/5/11) to trigger pyroptosis upon their activation [14,15].

One of the main mediators of PANoptosis in certain pathological states is Z-DNA binding protein 1 (ZBP1) [16]. It recognizes exogenous viral RNA or endogenous RNA via its Zα domain, thereby initiating the formation of the ZBP1-PANoptosome complex [17]. This complex formation promotes the cleavage of caspase-1/3/8, GSDMD/GSDME, and phosphorylation of MLKL, and ultimately coordinates the activation of the PANoptosis pathway [18]. Modulating the activation and function of ZBP1 could be a useful approach to regulating inflammatory pathogenesis.

The widely spread natural polyphenolic compound with the molecular formula C_16_H_18_O_9_, chlorogenic acid (CGA), is a compound present in the plant domain [19]. The compound has various biological activities, among which its notable antiviral and anti-inflammatory properties have attracted significant interest in more recent studies. As far as the antiviral effects are concerned, Ding confirmed that CGA could prevent the release of Influenza A virus (H1N1/H3N2) particles in vitro and showed high antiviral activity in mouse infection models [20]. In addition, Polyvinyl alcohol/poly(lactic-co-glycolic acid) nanoparticles loaded with CGA were also shown to suppress human coronavirus 229E in vitro [21]. The mechanism of action of CGA pertains to the anti-inflammatory potential of this compound, which entails regulation of the major pathways of inflammation and, therefore, prevents the production of pro-inflammatory cytokines, thus suppressing the inflammatory responses in a variety of disease models, such as cardiovascular diseases and inflammatory bowel disease [22,23]. The impact of CGA on CVB3-induced myocarditis, however, is not clear.

The present study was designed to test the hypothesis that CGA alleviates CVB3-induced myocarditis by promoting ZBP1 degradation via the ubiquitin–proteasome pathway, thereby inhibiting PANoptosis. To this end, we investigated the antiviral and anti-inflammatory effects of CGA both in vivo and in vitro, and dissected the stage of the viral life cycle targeted by CGA as well as the downstream cell death pathway involved.

## 2. Materials and Methods

### 2.1. Mice

Male Wu Laboratory Animals (Balb/c) (Fuzhou, China) were purchased at the age of 3 weeks with a body weight ranging between 15 and 20 g. All the experimental operations were granted by the Medical Ethics Committee of Huaqiao University (approval number: A2025140) and carried out following 3R-Refinement principles under free access to food and water after a 1-week acclimatization in controlled conditions (ambient temperature 23–25 °C, humidity 55–60%, 12 h/12 h light/dark cycle). Mice at 3 weeks old were induced with viral myocarditis by intraperitoneal injection of 10^6^ TCID50 CVB3 particles, and the control groups were intraperitoneally injected with the same volume of PBS. At 12 h post-infection, CGA (HY-N0055, MCE, Shanghai, China) was given (50 mg/kg or 100 mg/kg) by intraperitoneal injection every day. Each group consisted of 8 mice. This sample size was chosen based on previous publications in the CVB3-induced myocarditis field and is sufficient to detect statistically significant differences as shown in our results. Two extra mice per group were added to compensate for potential loss during the experiment. Body weights were taken on a daily basis. Hearts were quickly removed following euthanasia, washed, and kept at −80 °C until later RNA/protein extraction.

### 2.2. Cell Culture

Stored in liquid nitrogen, frozen HeLa cells (Procell, Wuhan, China) were thawed quickly using a 37 °C water bath. The thawed suspension was put into serum-free DMEM medium (11995, Solarbio, Beijing, China) and gently mixed and centrifuged at 1000 rpm for 5 min. The pellet containing the cells was resuspended in new HeLa-specific complete medium (CM-0101, Procell) after aspirating the supernatant that contained the cryopreservation agents. The cells were seeded into culture dishes with fresh medium and the cultures were kept in a humidified incubator at 37 °C with 5% CO_2_.

### 2.3. Virus

Professor Zhaohua Zhong (Department of Microbiology, Harbin Medical University, Harbin, China) kindly provided CVB3 Woodruff strain (GenBank: U57056.1) and EGFP-CVB3 [24]. The cells cultured to monolayer confluence were infected with 100 μL of the CVB3 viral stock. After 1 h adsorption under culture conditions, cells were kept at 37 °C in 5% CO_2_ till 75% cytopathic effect was detected. Three freeze–thaw cycles were thereafter applied to the infected cells in order to release viral particles. The lysate, which included CVB3 virions, was aliquoted and kept at −80 °C.

### 2.4. TCID50 Assay

HeLa cells in the logarithmic growth phase were seeded onto a 96-well plate. At the confluency of >85%, CVB3 virus suspension was diluted 10-fold (10^−1^ to 10^−10^) in eight replicates. 100 μL of each diluted viral inoculum was added to the respective wells and allowed to incubate at 37 °C for 2 h. After replacement with complete medium, cytopathic effects were recorded daily for 5 to 7 days. The Reed-Muench method was used to compute viral titers. All calculations were performed manually following the standard Reed-Muench formula.

### 2.5. Western Blotting

RIPA (R0010, Solarbio) lysis buffer was used to extract proteins from cells/tissues together with 1% protease and phosphatase inhibitors (P1260, Solarbio). After centrifugation at 10,000× *g* for 5 min at 4 °C, the supernatants were immediately collected to quantify protein concentration using the BCA assay (B6167, UElandy, Suzhou, China). Protein aliquots were denatured and stored at −20 °C. Samples were separated by 10% SDS-PAGE (S6182, UElandy) and transferred onto PVDF membranes. TBST that contained 5% non-fat milk was used to block membranes for 1 h at room temperature with agitation, after which the primary antibodies diluted in TBST were added to the membranes overnight at 4 °C. After three washes with TBST (12 min each), membranes were incubated with HRP-conjugated secondary antibodies for 1 h at room temperature. Membranes were then washed three times with TBST (12 min each), and protein bands were visualized using ECL substrate (S6009M, UElandy) and quantified with ImageJ software(version 1.54p, National Institutes of Health, Bethesda, MD, USA). The relative protein expression was adjusted to GAPDH (target protein/GAPDH band intensity ratio). Antibodies used: anti-RIP1 (AF7896, Beyotime, Shanghai, China), anti-RIP3 (AB316957, Abcam, Shanghai, China), anti-P-MLKL (AB187091, Abcam), anti-MLKL (21066-1-AP, Proteintech, Wuhan, China), cleaved caspase-1 (RM8397, Biodragon, Suzhou, China), cleaved caspase-3 (RM7270, Biodragon), GSDMD-N (BD-PT7991, Biodragon), P4D1 (3936, Cell Signaling, Shanghai, China), ZBP1 (BD-PN2476, Biodragon), anti-GAPDH (HRP-60004, Proteintech), Anti-mouse or rabbit secondary antibodies (SA00001, Proteintech). Anti-CVB3 3D was a gift from Prof. Zhaohua Zhong (Department of Microbiology, Harbin Medical University).

### 2.6. RT-qPCR

RNA-Quick Purification Kit (RN001, ESScience, Shanghai, China) was used to extract total RNA, and the Hiscript QRT SuperMix (RA103, Vazyme, Nanjing, China) was used to acquire cDNA. The Taq Pro Universal SYBR qPCR Master Mix (Q712, Vazyme) was used to carry out quantitative real-time PCR (qPCR). The amplification program was as follows: initial denaturation at 95 °C for 5 min, 40 cycles of denaturation at 94 °C for 30 s, annealing at 60 °C for 30 s, and extension at 72 °C for 30 s, and final extension at 95 °C for 15 min. Relative gene expression was normalized to GAPDH and calculated using the 2^−ΔΔCt^ method. Supplementary Table S1 includes the sequences of primers synthesized by Sangon Biotech (Shanghai, China) with the correct sequences provided.

### 2.7. Serum Levels of LDH and CK-MB

1.5 mL of whole blood from each mouse was placed into tubes and left at room temperature for 2 h to allow separation of serum and blood cells. The samples were then centrifuged at 5000× *g* for 10 min and serum was extracted and kept at −80 °C. Before the detection, serum samples were thawed at room temperature. Serum LDH and CK-MB levels were then determined using a fully automated biochemical analyzer (Mindray BS-240, Shenzhen, China) with commercially available kits according to the manufacturer’s instructions.

### 2.8. Viral Attachment Inhibition Assay

**Figure 3:**
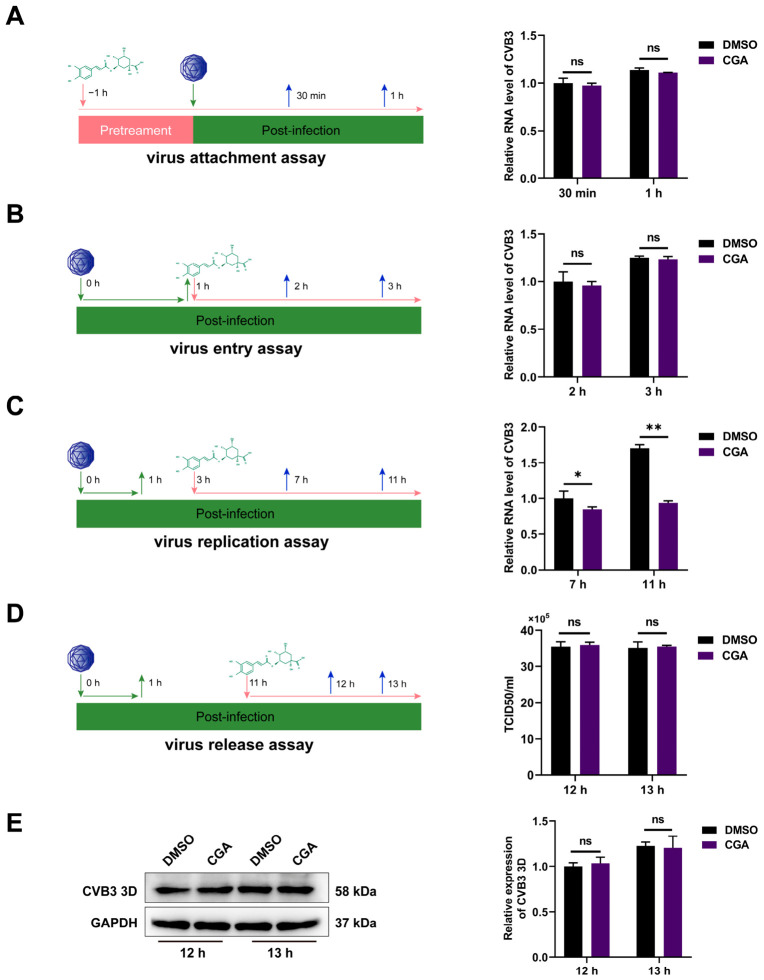
The antiviral effect of CGA is confined to the replication phase of CVB3. HeLa cells infected with CVB3 (MOI = 1)were treated with CGA during different phases of the viral life cycle. The viral genomic RNA was quantified by RT-qPCR. Meanwhile, the Western blotting analysis was performed to detect 3D protein at specified time points, using GAPDH as the loading control. (**A**) Virus attachment assay. (**B**) Virus entry assay. (**C**) Virus replication assay. (**D**,**E**) Virus release assay. The results represent the mean ± SD of three independent biological replicates. * *p* < 0.05; ** *p* < 0.01; ns, not significant.

The HeLa cells were cultivated in six-well plates and allowed to grow to confluence. Following pretreatment with CGA or DMSO at 37 °C for 1 h, cells were inoculated with CVB3 (MOI = 1) and incubated for 30 min or 1 h at 4 °C. Ice-cold PBS was used in order to remove unbound virus (Figure 3A). A total amount of RNA was isolated to quantify the levels of the CVB3 genomic RNA through RT-qPCR.

### 2.9. Viral Entry Inhibition Assay

Confluent HeLa monolayers in six-well plates were pre-chilled at 4 °C for 1 h. Cells were infected with CVB3 (MOI = 1) at 4 °C for 1 h, after which medium containing CGA or DMSO was added. Infection was allowed to proceed at 37 °C for 1 or 2 h before washing with PBS (Figure 3B). RT-qPCR was used to determine the levels of intracellular CVB3 genomic RNA.

### 2.10. Viral Replication Inhibition Assay

Confluent HeLa cells were exposed to CVB3 (MOI = 1) at 37 °C and incubated for 1 h. At 3 h post-infection, the medium was replaced with CGA- or DMSO-containing medium (Figure 3C). At 7 and 11 h post-infection (hpi), total RNA was extracted, and CVB3 genomic RNA was analyzed by RT-qPCR.

### 2.11. Viral Release Inhibition Assay

After incubating confluent HeLa monolayers with CVB3 (MOI = 1) at 37 °C for 1 h, the medium was changed to CGA- or DMSO-supplemented medium at 11 h post-infection. A further incubation of 1 h or 2 h at 37 °C yielded supernatants (Figure 3D). TCID50 was used to determine the viral titers, and Western blotting was used to assess intracellular levels of CVB3 3D.

### 2.12. Hematoxylin and Eosin (HE) Staining

The acquired myocardial tissues of each group were fixed, dehydrated, and embedded in paraffin. Slices (4 μm thick) were dewaxed, hydrated and stained with HE. Sequential dehydration, clearing, mounting and microscopic examination of stained sections were used in determining myocardial injury.

### 2.13. Cell Viability Assay

HeLa cells were seeded in 96-well plates at 1 × 10^5^ cells/mL and grown to confluence (37 °C, 5% CO_2_). After specific interventions, 10 μL of CCK-8 solution (CA1210, Solarbio) was added to each well. Absorbance was measured at 450 nm after placing the cells in a 1 h incubation (37 °C, 5% CO_2_). Statistical manipulations were employed to find viability.

### 2.14. siZBP1 Transfection

HeLa cells (5 × 10^5^ cells/well) were plated in six-well plates. When cells reached confluence, the medium was replaced with serum-free medium. Transfection was performed using siZBP1 (Sangon) (sense: 5′-GGAUGAGCAGUCCAAAGCA-3′; antisense: 5′-UGCUUUGGACUGCUCAUCC-3′) and an in vitro transfection reagent (KX0110049, Biodragon, Suzhou, China). The expression of proteins was determined 48 h after transfection.

### 2.15. Statistical Analysis

The statistical analyses were conducted with GraphPad Prism 9.0 (San Diego, CA, USA). The Shapiro–Wilk test was used to test normality, and the homogeneity of variances was tested through Levene’s test. For in vitro experiments, each experiment was independently repeated three times (biological replicates). Data are presented as the mean ± SD of the three biological replicates. One-way ANOVA was used for multiple group comparisons, followed by post hoc pairwise comparisons with Bonferroni adjustment when the overall F-test was significant. Each of the statistical tests was two-tailed and a *p* value < 0.05 was taken to be statistically significant.

## 3. Results

### 3.1. CGA Alleviates CVB3-Induced Myocarditis Through Concerted Antiviral and Anti-Inflammatory in Balb/c Mice

CGA is a ubiquitous phenylpropanoid phenolic acid and functions as an antiviral and anti-inflammatory bioactivator [25,26]. It is commonly found in traditional Chinese medicinal herbs such as *Eucommia ulmoides* and *Chrysanthemum* spp. However, its therapeutic activities on VMC triggered by CVB3 are yet to be determined. We utilized the anti-inflammatory and cardioprotective potential of CGA in a murine model of VMC caused by CVB3 under the hypothesis that CGA would be effective in reducing cardiac inflammation.

**Figure 1:**
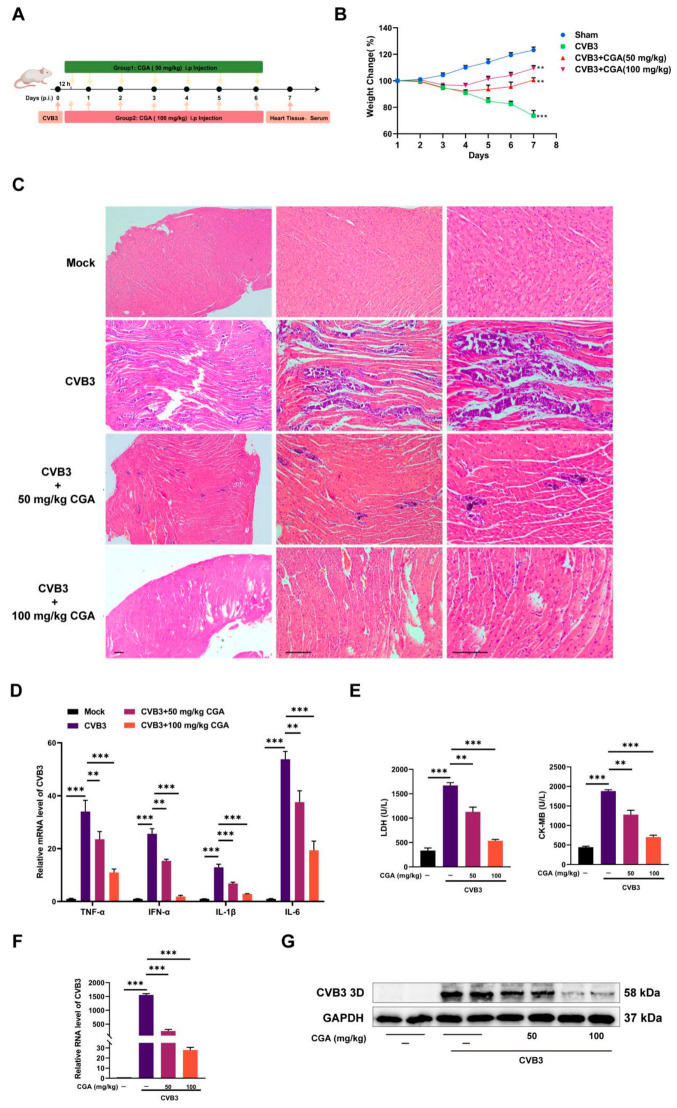
CGA Alleviates CVB3 Myocarditis via Dual Suppression of Viral Replication and Inflammation. (**A**) Experimental design. (**B**) The body weight of the mice was monitored daily for changes (n = 10 mice per group). (**C**) Histopathological assessment of cardiac lesions was performed on CGA-treated mice at 7 days post-infection (dpi) via HE staining (n = 5 hearts per group). Scale bar = 10 µm. (**D**) Expression of inflammatory cytokines was assessed by RT-qPCR amplification of myocardial tissue-derived total RNA. (**E**) LDH and CK-MB comparison between CGA-treated and control cohorts in serum. (**F**) CVB3 genomic RNA load measured by RT-qPCR using virus-specific primers in cardiac tissue-derived total RNA. (**G**) Comparative analysis of CVB3 3D protein levels in myocardial tissue by Western blotting under CGA-treated and untreated conditions. Data are presented as mean ± SD of three independent biological replicates. ** *p* < 0.01; *** *p* < 0.001.

Three-week-old Balb/c mice were intraperitoneally injected with 10^6^ TCID50 particles of CVB3. Mice were injected intraperitoneally with CGA (50 or 100 mg/kg) or PBS (as control) once daily for 7 days, starting at 12 hpi (Figure 1A). Mice were euthanized on day 7 post-infection, and cardiac tissue and serum were collected to assess histopathology and myocardial injury biomarkers. CVB3-infected mice exhibited significant body weight loss (27.3%), as shown in Figure 1B. This was reversed by both CGA dosage levels (50 and 100 mg/kg). Histopathological examination showed extensive myocardial structural damage and inflammatory cell infiltration in the CVB3 group (Figure 1C). In contrast, CGA intervention (50 and 100 mg/kg) preserved myocardial architecture and reduced inflammatory infiltration, indicating cardioprotection. This was verified by RT-qPCR, which showed that CGA treatment markedly reduced the myocardial concentrations of proinflammatory cytokines (TNF-α, IL-6, IL-1β, and IFN-α) (Figure 1D). Moreover, CGA therapy also lowered the serum levels of the cardiac injury markers CK-MB and LDH (Figure 1E). All these results reveal the anti-inflammatory effect of CGA in the CVB3-induced murine model.

The observed therapeutic activity of CGA in the VMC model suggested a possible effect on viral replication. To assess the impact of CGA on CVB3 replication, CVB3 3D expression and CVB3 genomic RNA levels were measured. CGA treatment significantly reduced viral genome levels and CVB3 3D expression in cardiac tissue, indicating inhibition of in vivo viral replication (Figure 1F,G). In summary, CGA reduces CVB3-induced myocardial damage through the combined effects of antiviral and anti-inflammatory processes.

### 3.2. Antiviral Activity of CGA Against CVB3 in HeLa Cells

**Figure 2:**
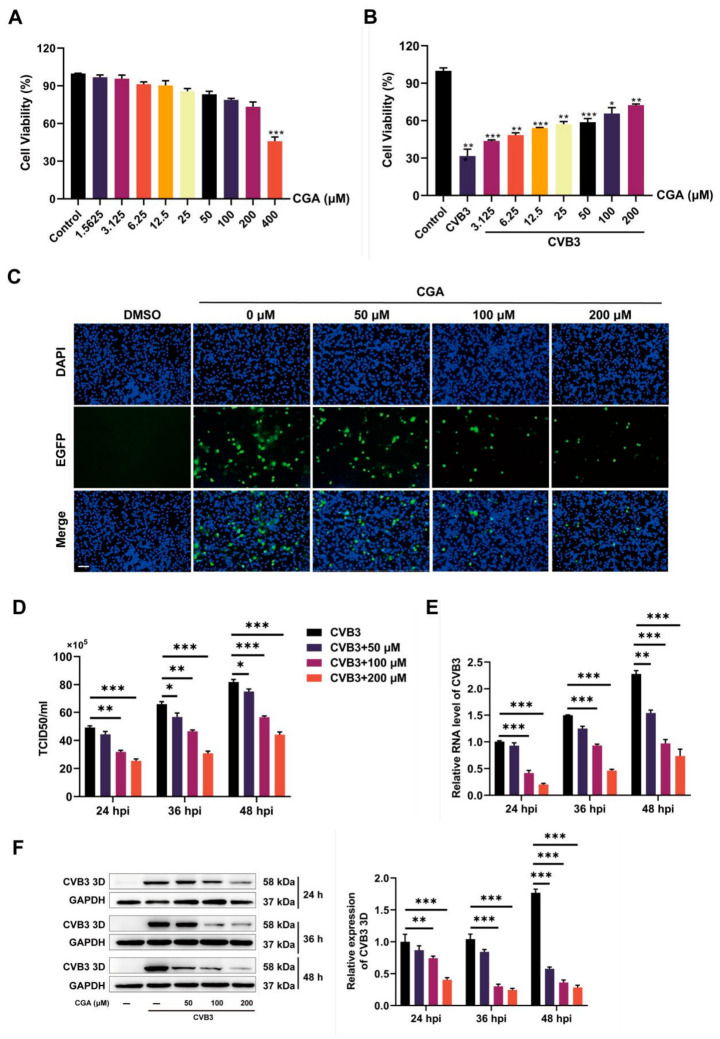
CGA inhibits CVB3 replication in HeLa cells in a dose- and time-dependent manner. (**A**) HeLa cells were incubated with various concentrations of CGA for 48 h, and cell viability was assessed using the CCK-8 assay. (**B**) HeLa cells were infected with CVB3 (MOI = 1) for 2 h and then treated with the indicated concentrations of CGA for 48 h. Cell viability was determined by the CCK-8 assay. (**C**) Fluorescence microscopy images of HeLa cells infected with EGFP-CVB3 at 24 hpi under three conditions: mock-infected, CVB3-infected, and CVB3-infected with CGA treatment. Scale bar = 10 µm. (**D**) Viral titers in CVB3 (MOI = 1)-infected HeLa cells were quantified by TCID50 assay at 24, 36, and 48 hpi. (**E**) HeLa cells infected with CVB3 (MOI = 1) and cultured in CGA-containing medium were harvested at 24, 36, and 48 hpi for total RNA extraction and RT-qPCR analysis. (**F**) Total protein from CVB3 (MOI = 1)-infected HeLa cells collected at the indicated time points was analyzed by Western blotting for CVB3 3D expression, with GAPDH as a loading control. Data are presented as mean ± SD of three independent biological replicates. * *p* < 0.05; ** *p* < 0.01; *** *p* < 0.001.

We then examined the antiviral effect of CGA in vitro. To assess the cytotoxicity of CGA in HeLa cells, cells were exposed to various concentrations of CGA for 48 h, and cell viability was measured using the CCK-8 kit. No noticeable cytotoxicity of CGA was detected even at the concentration of 200 μM, but a significant decrease in cell viability was observed at the concentration of 400 μM (Figure 2A). CGA treatment reduced the cytopathic effect of CVB3 in a concentration-dependent manner (0–200 μM) (Figure 2B). Based on these findings, further experiments were conducted using CGA at 50, 100, and 200 μM.

To further assess the antiviral properties of CGA, HeLa cells were infected with EGFP-CVB3, and viral replication was observed using fluorescence microscopy. Treatment with 100 μM and 200 μM CGA significantly reduced EGFP signal intensity (Figure 2C), indicating inhibition of viral propagation.

To measure cell-associated and extracellular virion production, viral titers were determined at various time points. Viral titers increased 1.7-fold between 24 and 48 hpi, as shown in Figure 2D. CGA treatment resulted in a significant, dose-dependent decrease in CVB3 titers, with reductions of 30.9% at 100 μM and 46.2% at 200 μM at 48 h. Western blot and RT-PCR revealed that CGA at 50–200 μM concurrently inhibited the level of CVB3 genomic RNA and the expression of CVB3 3D during all time periods in a dose- and time-dependent manner (Figure 2E,F). These results collectively indicate that CGA inhibits CVB3 replication in a dose- and time-dependent manner in vitro.

### 3.3. CGA Specifically Targets the Replication Stage of the CVB3 Life Cycle

To further examine the impact of CGA on CVB3 replication, we separately assessed its effects at each major stage of the viral life cycle. We used real-time RT-PCR to dynamically measure viral genomic load to determine the effects of CGA on the four stages: attachment, entry, replication, and release.

Pre-incubation with CGA did not significantly affect the binding capacity of CVB3 on the surface of HeLa cells as depicted in Figure 3A, implying that CGA had no interference effect on the initial viral adsorption step. Likewise, CGA present during the entry phase did not prevent the internalization of viruses into host cells (Figure 3B). Interestingly, CVB3 genomic copy number was significantly reduced when CGA was present during the replication phase; it was reduced by 44.9% in the presence of CGA compared to the DMSO control group, further demonstrating effective inhibition of viral replication by CGA (Figure 3C). On the other hand, we did not find statistically significant differences in viral titers or CVB3 3D expression levels between the CGA-treated and control groups during the release phase (*p* > 0.05) (Figure 3D,E). Overall, these results indicate that CGA prevents CVB3 infection by primarily affecting the viral replication phase, with no significant effects observed on attachment, entry, or release under the tested conditions.

### 3.4. CGA Inhibits CVB3-Induced PANoptosis by Downregulating ZBP1 Expression

**Figure 4:**
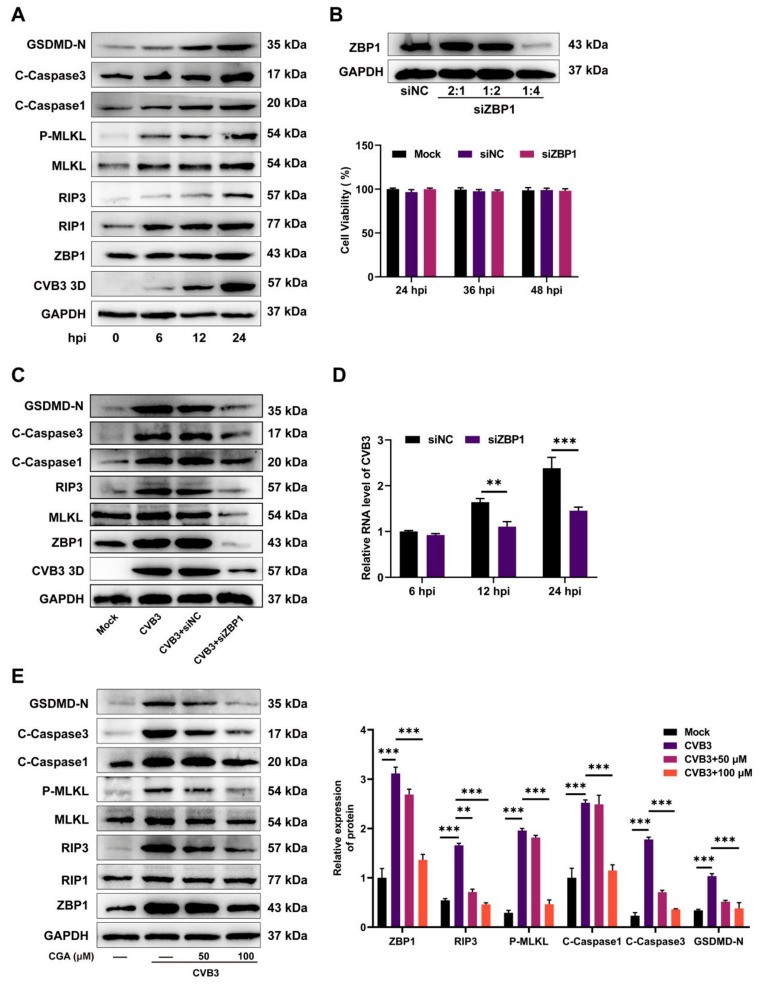
ZBP1 is a critical mediator of CVB3-induced cell death and a target of CGA. (**A**) Cells were infected with CVB3 (MOI = 1) and harvested at the indicated time points (0, 6, 12, and 24 hpi). Protein levels of ZBP1, C-Caspase3, RIP3, P-MLKL, C-Caspase1, and GSDMD-N were analyzed by Western blotting. GAPDH served as a loading control. (**B**) HeLa cells were transfected with siZBP1 at a ratio of 1:4. Knockdown efficiency was assessed by Western blotting, and cell viability was evaluated using the CCK-8 assay. (**C**) HeLa cells transfected with siNC or siZBP1 were infected with CVB3 (MOI = 1) for 24 h. Protein levels of CVB3 3D, ZBP1, C-Caspase3, RIP3, MLKL, C-Caspase1, and GSDMD-N were detected by Western blotting. (**D**) Total RNA was extracted from CVB3-infected HeLa cells following siNC or siZBP1 transfection, and CVB3 genomic RNA levels were quantified by RT-qPCR. (**E**) HeLa cells were infected with CVB3 (MOI = 1) and treated with CGA (50 or 100 μM) for 24 h. Protein levels of ZBP1, C-Caspase3, RIP3, MLKL, P-MLKL, C-Caspase1, and GSDMD-N were analyzed by Western blotting. Densitometric analysis of protein bands was performed using Image J software, and relative protein expression levels were normalized to GAPDH. The results represent the mean ± SD of three independent biological replicates. ** *p* < 0.01; *** *p* < 0.001.

In order to study the molecular process of CGA-mediated cardioprotective effect, we analyzed the behavior of ZBP1, which is an important sensor of innate immunity, in CVB3-infected HeLa cells. As shown in Figure 4A, there was a time-dependent increase in the expression of ZBP1 after CVB3 infection, with a significant increase seen at 12 and 24 hpi. At the same time, the levels of cleaved caspase-3 (C-Caspase3, an apoptosis marker), RIP3 and P-MLKL (necroptosis markers), as well as cleaved caspase-1 (C-Caspase1) and GSDMD-N (pyroptosis markers), gradually increased, indicating that CVB3 infection induces multidimensional programmed cell death.

To investigate the functional role of ZBP1 in CVB3 replication and CVB3-induced cell death, we performed siRNA-mediated knockdown of ZBP1 in HeLa cells. siRNA at a ratio of 1:4 resulted in effective silencing without apparent cytotoxicity, as confirmed by Western blotting and CCK-8 analysis (Figure 4B). Upon CVB3 infection, ZBP1 knockdown led to a marked decrease in CVB3 3D expression compared to the siNC control group. Furthermore, the infection-induced upregulation of C-Caspase3, RIP3, C-Caspase1, and GSDMD-N was significantly reduced in ZBP1-deficient cells (Figure 4C). Consistently, RT-qPCR showed that ZBP1 knockdown caused a substantial reduction in CVB3 genomic RNA levels (Figure 4D). Taken together, these findings indicate that ZBP1 promotes CVB3 replication and is required for the activation of various cell death processes during CVB3 infection.

Since CGA has antiviral effects as mentioned in the experiment above, we then examined whether CGA has an effect on ZBP1-mediated cell death. HeLa cells were infected with CVB3 and then treated with different doses of CGA (50 and 100 μM). Western blot was used to show that CVB3 infection strongly induced the expression of ZBP1, which was dose-dependently inhibited by CGA treatment. Meanwhile, CGA treatment inhibited the activation of apoptosis (C-Caspase3), necroptosis (RIP3, MLKL, P-MLKL), and pyroptosis (C-Caspase1, GSDMD-N) in a concentration-dependent manner (Figure 4E). These results indicate that CGA suppresses CVB3-induced programmed cell death, and this effect correlates with downregulation of ZBP1. However, whether this is a direct effect on ZBP1 or secondary to reduced viral replication remains to be determined.

### 3.5. CGA Promotes ZBP1 Degradation Through the Ubiquitin–Proteasome Pathway

**Figure 5:**
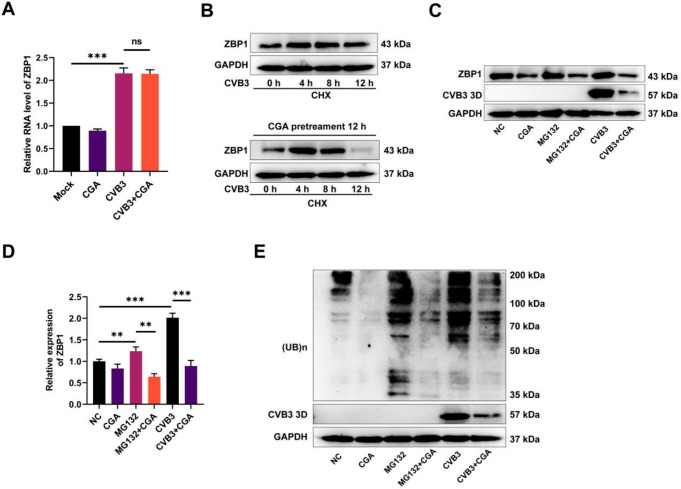
CGA downregulates ZBP1 by promoting its ubiquitin–proteasome-dependent degradation. (**A**) HeLa cells were infected with CVB3 (MOI = 1) and cultured for 24 h in media with or without CGA. Total RNA was extracted, and ZBP1 mRNA abundance was quantified by RT-qPCR. (**B**) Following 12 h pretreatment with CGA, HeLa cells were cultured in CHX (30 ng/mL)-containing medium for 12 h. Proteins were harvested at indicated post-infection time points for Western blotting analysis of ZBP1 expression. (**C**) HeLa cells were either treated with MG132 (30 μM) for 24 h or infected with CVB3 (MOI = 1) followed by CGA treatment for 24 h. Cellular lysates were subjected to Western blotting analysis. (**D**) Quantitative alterations in ZBP1 expression were calculated relative to GAPDH loading control. (**E**) Following identical treatments as in (**C**), cell lysates were analyzed by Western blotting. The results represent the mean ± SD of three independent biological replicates. ** *p* < 0.01; *** *p* < 0.001; ns, not significant.

Having demonstrated that CGA inhibits CVB3-induced PANoptosis by reducing ZBP1 expression, we next aimed to elucidate the molecular pathway underlying this regulation. To determine whether CGA affects ZBP1 at the transcriptional level, RT-qPCR was conducted on CVB3-infected HeLa cells treated with CGA for 24 h. As shown in Figure 5A, CVB3 infection significantly increased ZBP1 mRNA levels. However, CGA treatment did not reduce ZBP1 transcript levels compared to the infected group, indicating that the downregulation of ZBP1 by CGA does not occur primarily at the transcriptional level.

We then investigated the impact of CGA on the protein stability of ZBP1. To track the ZBP1 protein degradation dynamics in cells infected with CVB3, CHX chase assays were carried out. CGA pretreatment greatly accelerated the degradation of ZBP1 protein compared to untreated controls, indicating that CGA facilitates ZBP1 degradation at the post-translational level (Figure 5B).

Since the ubiquitin–proteasome system is the major pathway for regulated protein degradation, we examined its role in CGA-mediated ZBP1 degradation. Treatment with the proteasome inhibitor MG132 significantly increased ZBP1 protein levels in CVB3-infected cells, whereas co-treatment with CGA reversed this accumulation, supporting the involvement of proteasomal degradation in ZBP1 regulation (Figure 5C,D).

In order to further confirm the role of ubiquitin-dependent degradation, we analyzed global ubiquitination profiles. The accumulation of polyubiquitinated proteins occurred with potent MG132 treatment. CVB3 infection also led to the formation of a specific pattern of ubiquitinated proteins, which were of different molecular weight compared to those induced by MG132. Notably, the overall levels of ubiquitination were significantly decreased by CGA treatment (Figure 5E).

Together, these results indicate that CGA facilitates ZBP1 degradation via the ubiquitin–proteasome pathway, providing a mechanistic rationale for its suppressive effect on ZBP1 expression.

## 4. Discussion

VMC caused by CVB3 is a significant cause of sudden cardiac death and dilated cardiomyopathy among young people [27]. Though earlier research has identified various genes, proteins, and non-coding RNAs related to the pathogenesis of VMC, the underlying mechanisms are not yet fully understood, and effective antiviral treatments as well as specific clinical interventions are still lacking [28]. This study demonstrates that CGA possesses potent anti-inflammatory properties in an in vivo model of VMC. Moreover, CGA exhibited antiviral effects against CVB3 in both in vivo and in vitro studies and strongly inhibited the viral replication phase. Mechanistic studies demonstrated that the anti-inflammatory effects of CGA are largely attributed to its promotion of ZBP1 ubiquitination and subsequent degradation, thereby suppressing the activation of the ZBP1-mediated PANoptosis pathway.

Cell death is a significant host defense system against pathogens because it removes infections, but it can also cause cellular contents to be released and function as pathogen-associated molecular patterns, leading to intense inflammation [29]. It has been demonstrated by multiple studies that the apoptotic response in cardiomyocytes leads to further myocardial tissue damage in the pathogenesis of VMC, and cardiac functional recovery can be promoted through the inhibition of cardiomyocyte apoptotic pathways [30]. For example, Huang et al. observed increased MLKL phosphorylation in CVB3-infected HeLa cells, supporting the notion that CVB3 infection can trigger necroptosis [31]. Zeng et al. also found significantly higher protein levels of cleaved caspase-1 and GSDMD-N in myocardial tissues of VMC model mice compared to controls [32]. The major common molecular characteristics of pyroptosis, apoptosis, and necroptosis are incorporated in PANoptosis. It is worth mentioning that pharmacological or genetic inhibition of one programmed cell death pathway can cause the others to compensate, which can result in increased cell death and the development of the inflammatory response, eventually causing further aggravated tissue damage [33]. Thus, to inhibit PANoptosis, specific blockage of the upstream receptors of PANoptosome formation is necessary. Notably, loss of ZBP1 function or deletion of its Zα2 subdomain has been shown to significantly inhibit NLRP3 activation, reduce cleavage of caspase-3, caspase-8, and caspase-7, and block MLKL phosphorylation [17,34]. All these findings identify ZBP1 as a major upstream regulator of the PANoptosis pathway.

CGA is a natural bioactive compound that has shown considerable therapeutic benefits in reducing cardiovascular disorders, diabetes mellitus, hepatic and renal injuries, and tumorigenesis. Chlorogenic acid also exhibits diverse bioactivities, including colorectal cancer prevention via multi-target effects and epigenetic regulation of microRNAs, highlighting its therapeutic potential beyond viral myocarditis. Its anti-inflammatory effect is particularly notable, as it inhibits the NF-κB pathway to suppress pro-inflammatory factor expression and downregulates the p38 MAPK signaling pathway [35,36]. Additionally, CGA exhibits potent antioxidant effects by activating the nuclear factor erythroid 2-related factor 2 (Nrf2) pathway [37]. Further mechanistic studies have confirmed that CGA inhibits caspase activity and limits reactive oxygen species production, potentially providing effective protection against oxidative stress-induced tissue injury and apoptosis [38].

Considering this anti-inflammatory effect of CGA, this research tested its therapeutic effect on VMC induced by CVB3 infection. CGA administration (100 mg/kg/day) notably reduced myocardial tissue damage in CVB3-infected VMC mice without observable adverse effects. With respect to the current clinical evidence on CGA, high doses (2 g/day) or high intake of black tea (4 g/4 L/day) have been found to greatly augment the quantity of homocysteine in human plasma [39]. In contrast, a six-month CGA supplementation study (330 mg/day) indicated that there was a significant improvement in memory functionality among the participants [40]. Taken together, these safety data indicate that CGA is a promising therapeutic agent against CVB3 infection.

Previous research has demonstrated that CGA treatment during early infection with enterovirus 71 (EV71) in rhabdomyosarcoma cells significantly suppresses the expression of viral non-structural proteins and reduces mRNA levels [41]. Of note, CVB3 and EV71 both belong to the genus Enterovirus within the family Picornaviridae and possess conserved positive-sense, single-stranded RNA genomes. Although CGA has been shown to possess antiviral activity in EV71 and has been reported to have anti-inflammatory activity, we assumed that the antiviral ability of CGA can also act on CVB3 in the same genus of enterovirus. In order to test this hypothesis, we have estimated the antiviral effect of CGA with respect to CVB3. The findings showed that CGA treatment effectively prevented viral proliferation after infection. Initial mechanistic investigation revealed that CGA targets specific phases of the viral life cycle. Although we initially hypothesized that CGA would act during viral adsorption or entry, subsequent experiments showed that it did not significantly inhibit CVB3 entry into host cells but rather specifically targets the viral replication phase. However, the precise molecular target of CGA during the replication phase remains unclear. Potential candidates include the viral RNA-dependent RNA polymerase (3Dpol) or host replication factors that facilitate viral RNA synthesis. Future studies using in vitro polymerase activity assays, RNA binding assays, or chemical pull-down experiments are needed to identify whether CGA directly interacts with 3Dpol or modulates host replication machinery.

To further investigate the anti-inflammatory effect of CGA, we systematically analyzed the expression dynamics of PANoptosis-associated proteins in the present study. Consistent with previous findings, CVB3 infection markedly upregulated key apoptotic, necroptotic, and pyroptotic proteins. A synchronous and significant increase in ZBP1 expression, the central trigger of the PANoptosis pathway, was also observed. This observation implies that ZBP1 could be the major mediator of the PANoptotic process induced by CVB3. To confirm this hypothesis, we used RNA interference to knock down ZBP1. Western blot analysis clearly revealed that ZBP1 silencing markedly suppressed the upregulation of PANoptosis-related proteins induced by CVB3 infection. Crucially, this research also established that ZBP1-mediated activation of PANoptosis is specifically inhibited by means of CGA treatment. In a recent study, Huang et al. developed a PROTAC (proteolysis-targeting chimera) molecule using DNA aptamers and E3 ligase recruitment units [42]. Experimental validation demonstrated that this PROTAC effectively induced ZBP1 degradation and blocked the PANoptosis pathway, thereby alleviating influenza A virus-induced lung injury. Based on this study, we demonstrated that CGA enhances ZBP1 degradation via the ubiquitin–proteasome system.

It should be noted that the Traditional Chinese Medicine Systems Pharmacology Database and Analysis Platform reports that CGA has low oral bioavailability, only 11.93%. These findings indicate that CGA may suffer from limitations such as low intestinal absorption or excessive first-pass metabolism, making it difficult to achieve effective therapeutic concentrations. Previous research has demonstrated that CGA is broken down into caffeic acid and quinic acid in the intestine before absorption [43]. These aspects have greatly limited the use of CGA in the clinical setting, which is one of the issues that should be overcome in the future development of CGA-based therapeutics. Of note, Saleh et al. used electrospraying technology to encapsulate CGA within a polymer matrix and constructed a novel nano-delivery system [21]. This approach effectively enhances the oral bioavailability and antiviral capacity of CGA, thus offering a new platform to overcome the application barrier of CGA.

This study also has several limitations. First, the specific E3 ubiquitin ligase responsible for ZBP1 ubiquitination remains unknown, and it is unclear whether CGA promotes ZBP1 ubiquitination directly or indirectly via upstream signaling events. Potential candidates include RIP1, members of the TRIM family, or Parkin, all of which have been reported to be involved in the antiviral immune response. Second, all mechanistic PANoptosis data were obtained from HeLa cells, which are metabolically distinct from primary cardiomyocytes. Although HeLa cells are a standard model for CVB3 replication studies and our in vivo cardiac tissue data are consistent with the HeLa findings, future studies should validate the ZBP1-PANoptosis axis in human cardiac cell lines such as AC16 or in primary neonatal rat ventricular myocytes. Such validation will further solidify the cardiac relevance of the proposed mechanism. Third, it is unclear whether CGA interacts directly with ZBP1 or whether its effect is an indirect consequence of other ubiquitination-related interactions. Biophysical techniques, such as surface plasmon resonance and isothermal titration calorimetry, could be employed to assess direct binding. Fourth, the bidirectional relationship between ZBP1 and CVB3 replication complicates causal interpretation. The observation that siZBP1 reduces CVB3 replication suggests that ZBP1 itself promotes viral replication, creating a potential positive feedback loop. Therefore, when CGA reduces ZBP1 expression, the subsequent reduction in viral replication could secondarily lower PANoptosis markers. Conversely, the direct effect on ZBP1-mediated PANoptosis could also contribute. The current experimental design does not distinguish between these possibilities. Future studies using matched viral load conditions (e.g., infecting with different MOIs to achieve equivalent viral RNA levels) or ZBP1 overexpression rescue experiments are needed to resolve causal direction.

These limitations can be addressed in future studies by pursuing the following directions. The E3 ligases involved in ZBP1 ubiquitination upon CGA treatment could be identified by immunoprecipitation-mass spectrometry, followed by in vitro ubiquitination assays to confirm their functional roles. Cardiomyocyte-specific ZBP1 knockout mice would allow in vivo validation of the roles of ZBP1 and CGA in CVB3-induced myocarditis. Molecular docking and surface plasmon resonance studies might reveal direct binding between CGA and ZBP1, or identify E3 ligases that interact with CGA, potentially acting as molecular glues or allosteric modulators. Transcriptomic and proteomic studies on CGA-treated cells could provide more evidence on the signaling networks that are influenced by this substance and the implications of its pleiotropic effects. Lastly, preclinical pharmacokinetic and toxicological examination is necessary to create the basis for clinical translation.

## 5. Conclusions

**Figure 6:**
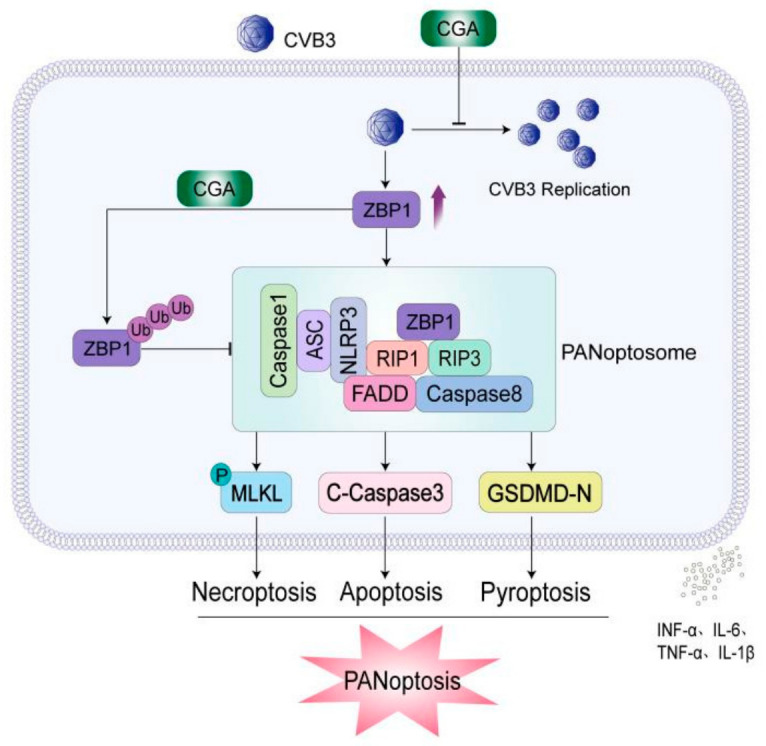
Scheme summarizing the inhibitory effect of CGA on CVB3-induced PANoptosis.

In summary, this study demonstrates that CGA alleviates CVB3-induced myocarditis through concerted antiviral and anti-inflammatory effects. In vivo, CGA ameliorates myocardial damage, reduces inflammatory cytokines and cardiac enzymes, and suppresses viral replication. In vitro mechanistic studies in HeLa cells show that CGA specifically blocks the replication phase of CVB3 and is associated with ZBP1 degradation involving the ubiquitin–proteasome pathway, correlating with reduced PANoptosis markers (Figure 6). These results position CGA as a potential therapeutic agent for VMC.

## Data Availability

The original contributions presented in this study are included in the article/Supplementary Materials. Further inquiries can be directed to the corresponding author.

## Supplementary Materials

The following supporting information can be downloaded at: https://www.mdpi.com/article/10.3390/microorganisms14061375/s1, Table S1: Gene primers used in RT-qPCR.
