# Supplementary material for: Chlorogenic Acid Ameliorates CVB3-Induced Viral Myocarditis by Suppressing Viral Replication and ZBP1-Mediated PANoptosis
Source: Microorganisms. 2026 Jun 21;14(6):1375. doi: 10.3390/microorganisms14061375 (PMC13305420; doi:10.3390/microorganisms14061375)
Supplement: Supplementary file 1 [file microorganisms-14-01375-s001.zip › microorganisms-4350523-supplementary.pdf]

Table S1 Gene primers used in RT-qPCR

Gene	Forward Primer	Reverse Primer
CVB3	GCACACACCCTCAAACCAGA	ATGAAACACGGACACCCAAAG
IL-1β (Mouse)	AAAGACCTCTATGCCAACACAGT	CTGACTTGGCAGAGGACAAAG
IL-6 (Mouse)	TAGTCCTTCCTACCCCAATTTCC	TTGGTCCTTAGCCACTACTTC
TNF-α (Mouse)	TCAAGTGGCATAGATGTGGAAGAA	TGGCTCTGCAGGATTTTCATG
IFN-α (Mouse)	GTCACTACGAATCGCACCTGATCACT	CCGATGTATAGACATTCCTCTTGG
GAPDH (Mouse)	AGGGCATCTTGGGCTACAC	CATACCAGGAAATGAGCTTGA
GAPDH (Human)	GCACCGTCAAGGCTGAGAAC	TGGTGAAGACGCCAGTGGA
ZBP1 (Human)	AACATGCAGCTACAATTCCAGA	AGTCTCGGTTCACATCTTTTGC
